# Genomic and Metabolic Features of an Unexpectedly Predominant, Thermophilic, Assistant Starter Microorganism, *Thermus thermophilus*, in Chinese Inner Mongolian Cheese

**DOI:** 10.3390/foods10122962

**Published:** 2021-12-02

**Authors:** Lin Zhu, Zhaozhi Hou, Xinyu Hu, Xu Liu, Tian Dai, Xinyu Wang, Chunlin Zeng, Yuan Wang, Caizheng Wang, Shujing Yang, Henglin Cui, Wei Wei

**Affiliations:** 1 School of Food and Biological Engineering, Jiangsu University, Zhenjiang 212013, China; zhulin19820402@ujs.edu.cn (L.Z.); 2211818038@stmail.ujs.edu.cn (Z.H.); 2211816006@stmail.ujs.edu.cn (X.H.); 3180906025@stmail.ujs.edu.cn (X.L.); 3180906022@stmail.ujs.edu.cn (T.D.); 3180906023@stmail.ujs.edu.cn (X.W.); 2211718010@stmail.ujs.edu.cn (C.Z.); 2211918033@stmail.ujs.edu.cn (Y.W.); 2222018071@stmail.ujs.edu.cn (C.W.); cuihenglin@ujs.edu.cn (H.C.); 2 School of Agricultural Engineering, Jiangsu University, Zhenjiang 212013, China; 2212016008@stmail.ujs.edu.cn

**Keywords:** Inner Mongolian cheese, high-throughput sequencing, *Thermus thermophilus*, lactose metabolism, organic acid generation

## Abstract

Inner Mongolian cheese is a traditional dairy product in China. It is produced without rennet, using naturally acidified milk that is simmered to achieve whey separation. In order to analyse the impact of simmering on the microbial community structure, high-throughput sequencing was performed to obtain bacterial 16S rRNA sequences from cheeses from the Ordos (ES), Ulanqab (WS), Horqin (KS) and Xilingol (XS) grasslands of Inner Mongolia. The relative abundance of an unexpected microorganism, *Thermus thermophilus*, ranged from 2% to 9%, which meant that its dominance was second only to that of lactic acid bacteria (LABs). Genome sequencing and fermentation validation were performed in *T. thermophilus* N-1 isolated from the Ordos, and it was determined that *T. thermophilus* N-1 could ingest and metabolise lactose in milk to produce lactate during the simmering process. *T. thermophilus* N-1 could also produce acetate, propionate, citrate and other organic acids through a unique acetate production pathway and a complete propionate production pathway and TCA cycle, which may affect texture and flavour development in Inner Mongolian cheese. Simultaneously, the large amount of citrate produced by *T. thermophilus* N-1 provides a necessary carbon source for continuous fermentation by LABs after the simmering step. Therefore, *T. thermophilus* N-1 contributes to cheese fermentation as a predominant, thermophilic, assistant starter microorganism unique to Chinese Inner Mongolian cheese.

## 1. Introduction

Cheese is a popular dairy product that is very important to the human diet and presents a variety of styles and flavours associated with its diverse geographical and cultural distribution [1]. Inner Mongolian cheese is the most common traditional cheese in China [2]. Inner Mongolian cheese is different from that of European and American cheeses. A simmering process involving naturally acidified milk is added during fermentation to replace the artificial addition of chymosin; thus, acidification and heating are used to promote the denaturation and solidification of casein in milk [3]. The simmering process can lead to the thermally imposed death of some microorganisms, similar to pasteurisation, and organic acids also exert some antibacterial effects [4]. Thus, the unique acidification and heating process involved in Inner Mongolian cheese production exerts a relevant microbial reduction. However, such acidification and heating provide appropriate conditions for the enrichment of some thermotolerant or thermophilic acidogenic microorganisms, with the temperature reaching 80~85 °C and pH reaching 4.5~6.5. Therefore, the simmering process in the preparation of Inner Mongolian cheese has the potential to shift the microbial community within the cheese, increasing the dominance of some thermotolerant or thermophilic acidogenic microorganisms in the microbial cheese community.

Lactic acid bacteria (LABs), such as *Lactococcus*, *Lactobacillus* and *Streptococcus* species, are used as starter microorganisms in cheese production and always account for more than 95% of the relative abundance of the microbial cheese community [5,6,7,8]. This is because these LABs can metabolise lactose, which was the absolute proportion of carbon source in milk, into glucose as the substrates of the Embden–Meyerhof–Parnas (EMP) metabolic pathway by β-galactosidase [9]. β-Galactosidase is an enzyme possessed by few microorganisms [10] but exists in some thermophilic microorganisms, such as *Thermus thermophilus* [11]. In our preliminary experiments involving the isolation of thermophilic bacteria, a strain of *T. thermophilus* N-1 was isolated from Inner Mongolian cheese samples (described in detail below), and the isolation of *T. thermophilus* was not reported in other cheese microbe-related studies. These results give us reason to believe that *T. thermophilus* with β-galactosidase may be selectively enriched in Inner Mongolian cheese in response to its distinctive simmering process.

The acidification of the milk used in Chinese Inner Mongolian cheese preparation is caused by organic acids produced mainly by LAB fermentation in milk [12]. These organic acids can acidify the milk and also act as flavours of Chinese Inner Mongolian cheese [13,14]. However, during simmering, LABs will be inhibited or even killed, and their acid production will stop [15]. Therefore, to reveal the role of *T. thermophilus* in Chinese Inner Mongolian cheese fermentation, high-throughput sequencing was performed to analyse the microbial community structure and dominant groups in cheese samples from different grasslands in Inner Mongolia. The whole-genome sequencing of the *T. thermophilus* N-1 isolate was performed, and the key enzyme genes related to lactose metabolism and organic acid production were annotated. The ability of *T. thermophilus* N-1 to metabolise lactose and produce a variety of organic acids was determined by comparing the metabolic pathways and fermentation products between the *T. thermophilus* N-1 strain and reference LABs. This study provides a reference for the large-scale, standardised production of Chinese Inner Mongolian cheese.

## 2. Materials and Methods

### 2.1. Chinese Inner Mongolian Cheese Samples and Food-Borne Pathogens Detection

We collected four kinds of Chinese Inner Mongolian cheese from different grasslands in the Inner Mongolia Autonomous Region of China, including Inner Mongolian cheese from Ordos (ES), Xilingol (XS), Horqin (KS) and Ulanqab (WS). The four kinds of cheese are cow cheese and prepared via almost the same traditional production process, in which after fresh milk is left to stand for 48 h, the acidified milk with the surface cream removed is simmered for approximately 4–6 h to completely separate the curds and whey [16]. After the whey is filtered out, the remaining curd is stirred and then placed in a mould for natural cooling for 24 h. The cooled curd is subsequently placed in a shaded area and dried for 7 days to obtain the final Inner Mongolian cheese [16].

Four pathogens were detected as soon as we collected the four cheese samples. *Salmonella* was detected qualitatively [17], and *Listeria monocytogenes* and *Escherichia coli* were detected quantitatively [17,18], but none of them was detected. *Staplococcus aureus* was detected by the Baird–Parker plate counting method [19], and the number of *Staphylococcus aureus* in the four cheese samples was in the range of 10–50 CFU/g, which was less than the safety standard required for dairy products [20].

### 2.2. High-Throughput Sequencing Based on the Bacterial 16S rRNA Genes of Cheese Samples

Microbial genomic DNA was extracted from the cheese samples using the PoweFood Microbial DNA Isolation Kit (MoBio Laboratories Inc., Solana Beach, CA, USA). One gram of cheese was combined with 9 mL of 2% trisodium citrate and homogenised before DNA extraction [21]. The DNA samples were amplified by PCR using the universal prokaryotic primers 515F and 806R for the V4 region of the 16S rRNA gene [21]. The 16S rRNA V4 amplicons were sequenced on an Illumina MiSeq platform according to the paired-end protocol, and the average read length was 252 bp. The quality control procedures and the determination and annotation of operational taxonomic units (OTUs) were performed according to the descriptions of Zeng et al. [21].

### 2.3. Strain Isolation and Genome DNA Extraction

The strain *T. thermophilus* N-1 was isolated from the cheese sample from the Ordos by using ML medium that contains (g/L): 10.0 tryptone, 10.0 NaCl, 5.0 yeast extract (adjusted to pH = 7.0), and incubated at 80 °C. The *T. thermophilus* N-1 strain was enriched in CM0828 liquid medium at 80 °C until the midlogarithmic phase was reached, and the cells were then centrifuged for collection. The genomic DNA of *T. thermophilus* N-1 was extracted by using the genomic DNA extraction kit (CW0552; Beijing ComWin Biotech Co., Ltd., Beijing, China). The quality assessment of the purified DNA was performed by agarose gel electrophoresis, and its quantification was performed on a Qubit 2.0 system.

### 2.4. Genome Sequencing and Assembly

The genomic DNA of *T. thermophilus* N-1 was sent to Beijing Novogene Bioinformatics Technology Co., Ltd. (Beijing, China) and sequenced by using Pacific Biosciences RS II single-molecule real-time (SMRT) sequencing technology and Illumina high-throughput sequencing technology. Sequence reads were generated from the 10 kb SMRT Bell library and a 350 bp library. After subread filtering of the raw data from the PacBio RS II and Illumina PE150 sequencers, low-quality reads were filtered out by SMRT Link 5.0.1 [22,23], and assembly was performed. The complete genome sequence of *T. thermophilus* N-1 was submitted to the GenBank database under accession number CP082323-CP082325.

### 2.5. Genome Component Prediction

GeneMarkS [24] (http://topaz.gatech.edu/GeneMark/, accessed on May 2020) was used to predict the coding genes of the sequenced genome. RepeatMasker [25] was used to predict scattered repeats, and Tandem Repeats Finder (TRF) Version 4.07b [26] was used to search for tandem repeats in the DNA sequences. tRNAs were predicted by using tRNAscan-SE [27]. RNAmmer [28] was used to predict rRNAs. First, we performed annotation via comparison with the Rfam database [29], and we then used the cmsearch program (Version 1.1rc4, with default parameter) to determine the final sRNA gene set [30]. Island Island-DIOMB [31] in the sample genome were predicted with phiSpy (Version 2.3) [32]. CRISPRdigger [33] was used for CRISPR prediction of the sample genome.

### 2.6. Genome Annotation

Five databases were used to predict gene functions. They were the Gene Ontology (GO) [34], Kyoto Encyclopedia of Genes and Genomes (KEGG) [35], Clusters of Orthologous Groups (COG) [36], and the Non-Redundant Protein Database databases (NR) [37]. Whole-genome BLAST searches (E-value less than 1e-5, minimal alignment length percentage greater than 40%) were performed against the above five databases. Additionally, secondary metabolism gene clusters were analysed by using antiSMASH [38]. Carbohydrate-active enzymes were predicted in the Carbohydrate-Active EnZymes Database [39]. The complete genomes of some reported LABs isolated from different cheeses, including *Lactobacillus plantarum* 10CH, *Lactococcus lactis* SD96, *Streptococcus thermophilus* NCTC 12958, *Leuconostoc mesenteroides* DSM 20484, *Lactobacillus paracasei* NJ and *Lactobacillus fermentum* MTCC 25067, were obtained from the NCBI Database to perform the comparative genome annotation of *T. thermophilus* N-1 (https://www.ncbi.nlm.nih.gov/, accessed on May 2020). The sources and accession numbers of the isolates are given in Table S1.

### 2.7. Verification of the Milk Fermentation Ability and Characteristics of T. thermophilus N-1

In order to clarify the role of *T. thermophilus* in the preparation of traditional Chinese cheese, the *T. thermophilus* N-1 strain and the *Lactobacillus fermentum* 11-1S4 strain (the dominant LAB species isolated from the same Ordos cheese samples as *T. thermophilus* N-1) were used to ferment the milk. In order to obtain *L*. *fermentum* 11-1S4 and *T*. *thermophilus* N-1 with similar cell numbers, the culture endpoint with an OD_600_ value of 0.3 was determined based on pre-experiment. The OD_600_ value of culture medium inoculated with *L*. *fermentum* 11-1S4 and *T*. *thermophilus* N-1 was reached 0.3 by culturing for 4 h and 6 h, respectively. At this time, the number of *L*. *fermentum* 11-1S4 and *T*. *thermophilus* N-1 were determined to be 6.2 × 10^7^ CFU/mL and 2.5 × 10^7^ CFU/mL, respectively, by the dilution plate counting method, and 1 mL of *L*. *fermentum* 11-1S4 and *T*. *thermophilus* N-1 solution was centrifuged to obtain bacterial precipitation. Then, the bacterial precipitations of *L*. *fermentum* 11-1S4 or *T*. *thermophilus* N-1 were inoculated into 100 mL sterilised milk to determine their milk fermentation ability at the test temperature. We set up six treatments involving different inoculation and culture methods: (a) uninoculated sterilised milk, (b) sterilised milk inoculated with *L. fermentum* 11-1S4 and cultured at 80 °C for 24 h, (c) sterilised milk inoculated with *T. thermophilus* N-1 and cultured at 37 °C for 24 h, (d) sterilised milk inoculated with *L. fermentum* 11-1S4 and cultured at 37 °C for 24 h, (e) sterilised milk inoculated with *T. thermophilus* N-1 and cultured at 80 °C for 24 h and (f) treatment e inoculated with *L. fermentum* 11-1S4 and cultured at 37 °C for 24 h. After fermentation, the CFU, pH values, lactose contents and organic acid types and concentrations were measured. pH was measured by using a pH meter (pHS-3TC; Shanghai Tianda Instrument Co., Ltd., Shanghai, China). The lactose content was measured using the HPLC method [40]. Organic acids, including oxalic acid, tartaric acid, malic acid, fumaric acid, lactic acid, acetic acid, citric acid, succinic acid and propionic acid, were determined by high-performance liquid chromatography (HPLC) (Thermo Fisher Technologies, Waltham, MA, USA). The analysis conditions referred to Akalin et al. [14].

## 3. Results

### 3.1. Relative Abundance of T. thermophilus in the Bacterial Community of Chinese Inner Mongolian Cheese

**Figure 1:**
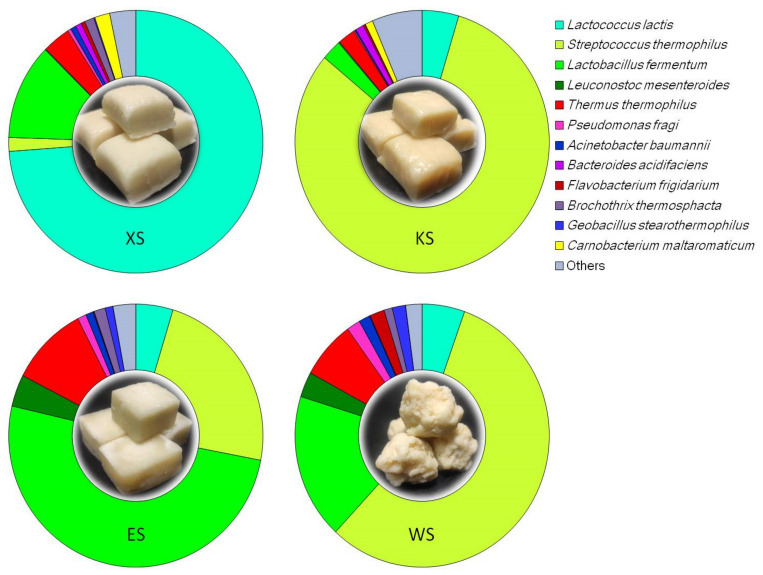
Dominant bacterial species with a relative abundance greater than 1% in different cheese samples. The sizes of the different-coloured areas in the rings represent the relative abundance of different dominant species. Cheese samples are shown in the middle of the rings. ES, cheese sample from the Ordos grassland; XS, cheese sample from the Xilingol grassland; KS, cheese sample from the Horqin grassland; WS, cheese sample from the Ulanqab grassland.

Based on high-throughput sequencing, the bacterial 16S rRNA genes of four tested cheese samples from Inner Mongolia were sequenced. The sequencing libraries of the four samples showed qualified library coverage (Table 1). Based on sequence alignment results, different OTU numbers were obtained from the four tested samples, ranging from 389 to 646. The annotation results of these OTUs showed that LABs, including *Streptococcus thermophilus*, *Lactococcus lactis*, *Lactobacillus fermentum* and *Leuconostoc mesenteroides* were dominant in the bacterial communities of the four tested cheeses, and their total relative abundance ranged from 86.9% to 90.9% (Figure 1). *T. thermophilus* was the only species other than LABs with a relative abundance of more than 1% in all samples, and its relative abundance ranged from 2.2% to 9.9% (Figure 1). The relative abundance of *T. thermophilus* in the cheese samples from the Ordos was 9.9%. Therefore, the *T. thermophilus* N-1 strain was isolated from the cheese samples of Ordos at 80 °C via a method based on the selective ML medium. This N-1 strain was used as a representative strain of *T. thermophilus* for subsequent genome analysis and fermentation validation experiments.

### 3.2. General Genome Features of T. thermophilus N-1

**Figure 2:**
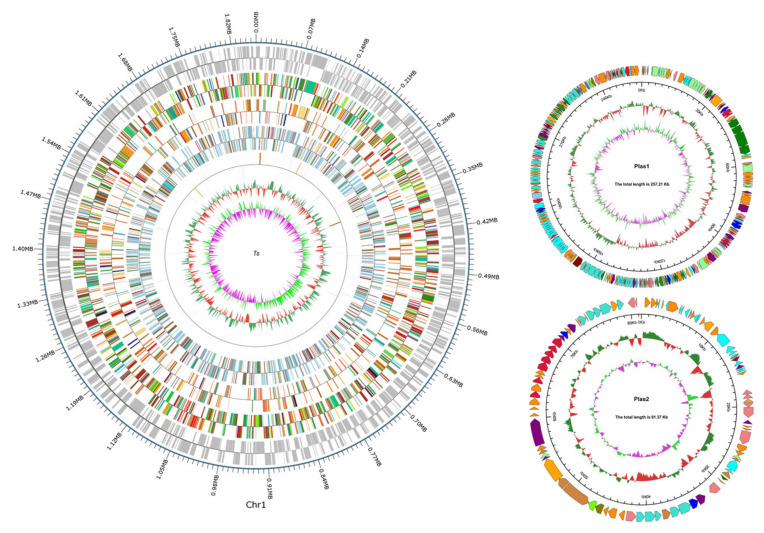
Genome map of *T. thermophilus* N-1. From the outer cycle to the inner cycle, ring 1 represents the total base pairs and the position coordinates of the genomic sequences; ring 2 shows the coding sequences; rings 3, 4 and 5 represent functional annotation results coloured according to their COG, KEGG and GO origins, respectively; ring 6 represents noncoding RNA (ncRNA) genes; ring 7 represents GC contents; and ring 8 represents the GC skew value distribution. The image was created by using the software Circos. For the two plasmids of *T. thermophilus* N-1, circles 1–4 (from outermost to innermost) represent the COG functional annotation classifications of genes (a clockwise arrow indicates the positive coding strand), genomic sequence position coordinates, genomic GC contents and the genomic GC skew value distribution.

The sequencing of *T. thermophilus* N-1 generated 98,992 subreads and 655,471,669 base pairs, with 298-fold genome coverage. One contig without gaps was obtained after assembling the filtered low-quality reads with SMRT Link v5.0.1. A complete genome map of *T. thermophilus* N-1 was drawn (Figure 2). The complete genome was 2,194,207 bp in length and contained a circular chromosome with a G + C content of 69.51% and two circular plasmids with G + C contents of 69.33% and 67.95% (Table 2). Six rRNA operons, 48 tRNA genes, 11 genomics islands, 10 clustered regularly interspersed short palindromic repeats (CRISPRs), and 2328 protein-coding genes (CDSs) were identified. Twenty-one interspersed repetitive sequences, including 11 long terminal repeats (LTRs), 3 DNA transposons, 2 long interspersed repeated segments (LINEs), 4 short interspersed repeated segments (SINEs) and 1 unknown sequence, were identified. Additionally, 139 tandem repeat sequences (repeat size 9–228 bp), including 128 minisatellite DNA sequences (repeat size 11–56 bp), were found.

### 3.3. Functional Annotation of T. thermophilus N-1

**Figure 3:**
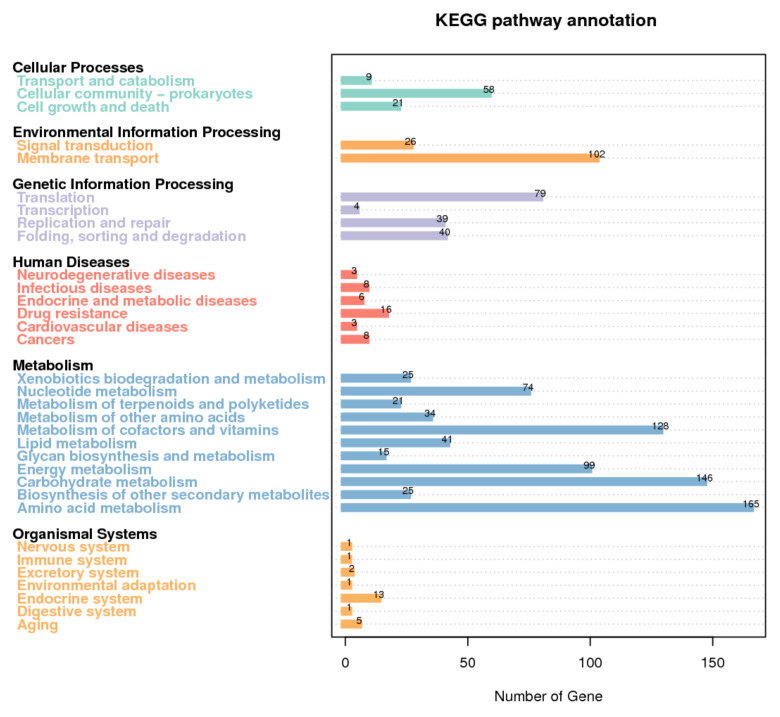
KEGG annotation of *T. thermophilus* N-1 based on pathway type and gene number.

In order to gain insight into the gene functions of *T. thermophilus* N-1, the KEGG and COG databases were used to functionally annotate the genome. The genome annotation results revealed that 2251 CDSs could be assigned to the KEGG database (Figure 3) and that 1918 CDSs could be assigned to the COG database (Figure S1). Based on the KEGG pathway annotations, 1522 proteins were associated with 178 KEGG metabolic pathways, which was greater than the number of proteins associated with other pathways. Among the 773 genes encoding proteins associated with metabolic pathways, 52% were related to the metabolism of nutrients (e.g., lipids, proteins and carbohydrates), including 41 genes associated with lipid metabolism, 15 genes associated with glycan biosynthesis and metabolism, 146 genes associated with carbohydrate metabolism, 165 genes associated with amino acid metabolism and 34 genes associated with the metabolism of other amino acids (Figure 3). Based on COG function classification, 1555 identified coding proteins were classified into 25 functional categories. Among the 810 genes encoding proteins associated with metabolic pathways, 55% were related to the metabolism of nutrients (e.g., lipids, proteins and carbohydrates), including 99 genes associated with lipid transport and metabolism, 144 genes associated with carbohydrate transport and metabolism and 204 genes associated with amino acid transport and metabolism (Figure S1). These results indicated that *T. thermophilus* N-1 has the ability to metabolise proteins, lipids and carbohydrates, which may play a role in changing the nutritional composition and flavour during the simmering process in Chinese Inner Mongolian cheese fermentation.

### 3.4. Distribution of Enzymes Associated with Lactose Metabolism

**Figure 4:**
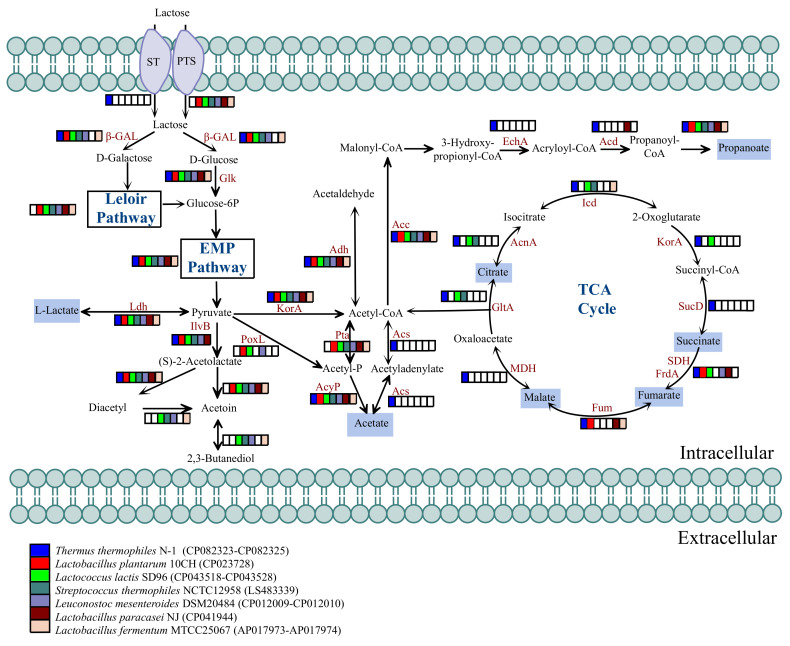
Predicted metabolic network of lactose metabolism and organic acid production in *T. thermophilus* N-1. EMP, Embden–Meyerhof–Parnas pathway; TCA cycle, tricarboxylic acid cycle. ST, sugar transporter(glycoside/pentoside/hexuronide: cation symporter, GPH family); PTS, phosphoenolpyruvate phosphotransferase system; Glc, glucose uptake permease; β-GAL, beta-galactosidase; Glk, glucokinase; Ldh, L-lactate dehydrogenase; IlvB, acetolactate synthase; PoxL, pyruvate oxidase; KorA, 2-oxoglutarate/2-oxoacid ferredoxin oxidoreductase; Adh, acetaldehyde dehydrogenase; Acs, acetyl-CoA synthetase; Acc, acetyl-CoA carboxylase/biotin carboxylase; EchA, enoyl-CoA hydratase; Acd, acyl-CoA dehydrogenase; GltA, citrate synthase; AcnA, aconitate hydratase; Icd, isocitrate dehydrogenase; KorA, 2-oxoglutarate/2-oxoacid ferredoxin oxidoreductase subunit alpha; SucD, succinyl-CoA synthetase; SDH, succinate dehydrogenase; FrdA, fumarate reductase; Fum, fumarate hydratase; MDH, malate dehydrogenase.

Based on the genome annotation results of *T. thermophilus* N-1 obtained from the KEGG database, we screened the genes encoding core enzymes related to lactose hydrolysis and predicted the metabolic pathway map (Figure 4). *T. thermophilus* N-1 exhibited genes encoding a putative sugar transporter and β-galactosidase, which suggested that extracellular lactose could be transported intracellularly and decomposed to glucose and galactose by β-galactosidase. This lactose metabolism process suggests that *T. thermophilus* N-1 could also use the lactose in raw milk as a carbon source for normal growth, similar to LABs. Therefore, *T. thermophilus* N-1 can grow in milk by metabolising lactose during the simmering process in Chinese Inner Mongolian cheese fermentation, which makes *Thermus* among the predominant genera in the bacterial cheese community, similar to LABs.

Subsequently, genomic data were collected from six different LABs isolated from different cheeses, including *Lactobacillus plantarum* 10CH, *Lactococcus lactis* SD96, *Streptococcus thermophilus* NCTC 12958, *Leuconostoc mesenteroides* DSM 20484, *Lactobacillus paracasei* NJ and *Lactobacillus fermentum* MTCC 25067, and the genes related to milk fermentation were annotated (Figure 4). We found that these reference LABs all harboured an enzyme not found in *T. thermophilus* N-1 that can metabolise D-galactose produced via lactose metabolism into glucose-6P through the Leloir Pathway, after which this product enters the EMP Pathway. These results showed that the LABs in Chinese Inner Mongolian cheese might have more available carbon source substrates than *T. thermophilus* N-1 for metabolism under conditions in which the same amount of lactose is metabolised.

### 3.5. Distribution of Enzymes Associated with Organic Acid Generation

Further genome annotation results showed that the previously produced glucose could be transformed into pyruvate through the EMP pathway, after which pyruvate is metabolised into the various organic acids, which could accelerate the coagulation of milk by reducing the pH and play a role as flavouring substances (Figure 4). Lactate could be generated by L-lactate dehydrogenase (Ldh), as observed in the LABs we collected through homolactic fermentation (Figure 4). In contrast to the acetate production pathway of reference LABs, *T. thermophilus* N-1 may produce acetate from acetyladenylate via the action of acetyl-CoA synthetase (Acs) (Figure 4). *T. thermophilus* N-1 has a complete propanoate production pathway, which is not present in all reference LABs and can convert acetyl-CoA into propanoate (Figure 4). Additionally, *T. thermophilus* N-1 exhibits a complete TCA cycle pathway, which is also not found in reference LABs and can generate a variety of organic acids, including citrate, malate, fumarate and succinate (Figure 4). These results indicated that *T. thermophilus* N-1 might be able to produce a variety of organic acids similar to cheese LABs, which can promote the coagulation and acid flavour formation of cheese. Therefore, *T. thermophilus* may play a similar role to LABs in organic acid generation during the simmering process in the fermentation of Chinese Inner Mongolian cheese.

### 3.6. Validation of T. thermophilus N-1 in Milk during Fermentation

**Figure 5:**
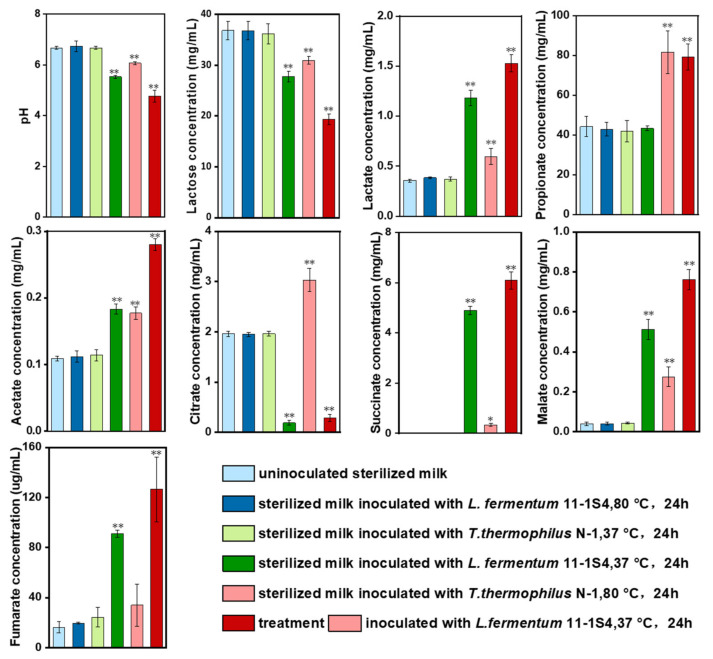
Validation of *T. thermophilus* N-1 fermentation in milk, including the pH change, lactose metabolism, and organic acid generation. *, ** indicate significant difference at *p* < 0.05 and *p* < 0.01 compared with the uninoculated sterilised milk.

To verify the lactose metabolic pathway and four major organic acid-producing metabolic pathways predicted via whole-genome sequencing, we carried out culture experiments of *T. thermophilus* N-1. Simultaneously, a strain 11-1S4 belonging to *Lactobacillus fermentum*, the most dominant species in Ordos cheese samples (Figure 1), was used as a representative of LABs for fermentation experiments (Figure 5). The CFU number of milk samples inoculated with *L. fermentum* 11-1S4 after 24 h of culture at 37 °C and 80 °C was 9.5 × 10^10^ CFU/mL and 3.0 × 10^5^ CFU/mL, respectively. The CFU number of milk samples inoculated with *T. thermophilus* N-1 after 24 h of culture at 37 °C and 80 °C was 1.0 × 10^5^ CFU/mL and 1.2 × 10^10^ CFU/mL, respectively. After 24 h at 37 °C, the pH and the lactose and organic acid contents of the milk inoculated with *T. thermophilus* N-1 were almost the same as those of the control, as observed in the milk inoculated with *L. fermentum* 11-1S4 at 80 °C (Figure 5). However, the pH and lactose content of the milk inoculated with *T. thermophilus* N-1 decreased significantly after 24 h at 80 °C (*p* < 0.05). This was consistent with the changing trend observed in milk inoculated with *L. fermentum* 11-1S4 at 37 °C, although the downward trend was not as obvious as that associated with *L. fermentum* 11-1S4. These results showed that *Thermus* could replace LABs (whose metabolism is inactivated by the simmering process in Chinese Inner Mongolian cheese fermentation), metabolise lactose and produce moderate amounts of organic acids, thus playing a potential role as a starter microorganism.

The contents of lactate, acetate, malate and succinate in milk inoculated with *T. thermophilus* N-1 at 80 °C for 24 h were significantly higher than those under the control treatment (*p* < 0.05), but the degree of the increase was smaller than that in milk inoculated with *L. fermentum* 11-1S4 (Figure 5). Simultaneously, the content of propionate, which *L. fermentum* 11-1S4 cannot produce, was also increased significantly in milk (*p* < 0.05). It should be noted that the content of citrate in milk increased significantly but was almost completely metabolised in milk inoculated with *L. fermentum* 11-1S4 (Figure 5). These results indicated that *T. thermophilus* N-1 has the ability to produce organic acids similarly to *L. fermentum* 11-1S4. Although its ability to produce organic acids is weaker than that of *L. fermentum* 11-1S4, the variety of organic acids it produces is more diverse, and it can provide abundant citrate, which is necessary for *L. fermentum* 11-1S4 metabolism, to assist LABs in cheese fermentation.

After the inoculation of *L. fermentum* 11-1S4 and fermentation for 24 h, the lactose content of 1 mL of milk decreased by 9.1 mg, the pH value decreased by 1.1, and the lactate, succinate, malate and fumarate contents increased by 827.4, 4875.2, 473.3 and 74.9 μg, respectively (Figure 5). When *L. fermentum* 11-1S4 was inoculated into milk samples that were inoculated with *T. thermophilus* N-1 and fermented for 24 h, the lactose content of 1 mL of milk decreased by 11.5 mg, the pH value decreased by 1.3, and the lactate, succinate, malate and fumarate contents increased by 932.5, 5761.6, 486.8 and 92.5 μg, respectively (Figure 5). The above series of values of milk inoculated with *T. thermophilus* N-1 and *L. fermentum* 11-1S4 always exceed those of inoculated with only *L. fermentum* 11-1S4. These results indicate that the lactose metabolism, organic acid production and pH-reducing abilities of *L. fermentum* 11-1S4 were enhanced through the action of *T. thermophilus* N-1, which may be the result of the promotion of *L. fermentum* 11-1S4 metabolism by citrate production by *T. thermophilus* N-1. All the above results suggest that *T. thermophilus* N-1 can metabolise lactose and decrease pH levels, similar to LABs, in the simmering process of Chinese Inner Mongolian cheese, and its products can promote the growth and fermentation of LABs in milk after this simmering process.

## 4. Discussion

Chinese Inner Mongolian cheese is produced without adding any starter microorganisms or exogenous chymosin; instead, a unique simmering and stirring process is added to cause the milk to coagulate. The simmering process conducted at a high temperature of approximately 80 °C provides a suitable enrichment temperature for *Thermus*, a thermophilic bacterial genus. Simultaneously, through genome annotation and fermentation experiments, we successfully verified that the *T. thermophilus* N-1 strain might transport lactose from milk into cells by using putative lactose transporters (STs) that are different from those of reference LABs and can metabolise lactose by using a β-galactosidase (β-GAL) that is the same as that of reference LABs [41,42]. Although *T. thermophilus* N-1 lacks the complete D-galactose Leioir pathway and its ability to produce D-glucose by metabolising lactose is weaker than that of LABs, the high temperature of the simmering process used in China Inner Mongolian cheese fermentation means that the lactose metabolism function of *T. thermophilus* N-1 cannot be replaced by LABs.

In addition to the lactate produced by lactose metabolism, *T. thermophilus* N-1 plays an important role in the production of other organic acids. For example, *T. thermophilus* N-1 exhibits an acetate synthesis pathway dominated by acetyl-CoA synthetase (Acs), which is not found in LABs; *T. thermophilus* N-1 has a complete TCA cycle pathway, which is also lacking in LABs [43], and can thus produce citrate, malate, fumarate and succinate; and *T. thermophilus* N-1 has a complete propanoate synthesis pathway, which is not observed in LABs. It was reported that lactate, acetate, propanoate and citrate could play important roles as flavouring substances in the formation of characteristic flavours in cheese [44,45,46]. Some of the inorganic acid products produced by organic acid metabolism are also sources of special flavours in cheese. For example, we screened the *adh* gene, which encodes an enzyme that converts acetyl-CoA to acetaldehyde, a volatile aromatic substance produced during hetero-alcoholic fermentation in acetate generation [47]. We also screened the gene encoding the enzyme that converts acetolactate to diacetyl, a buttery substance produced during acetolactate metabolism [48]. Therefore, the metabolism of organic acids by the *Thermus* genus might contribute to the formation of special flavours in Chinese Inner Mongolian cheese. Additionally, salt plays an important role in cheese-making, including preservation, contributions to flavours and acting as a source of dietary sodium [49]; salt contents in China Inner Mongolian cheese range from 0.1 to 0.7%. *T. thermophilus* can grow in the salt concentration range of 0~4% [50], which is also sufficient to meet the salt addition demand in cheese production [51].

It was reported that citrate is an essential carbon source substrate used by LABs in milk fermentation, and various fermentation pathways are related to citrate metabolism [52]. In our fermentation validation experiment, it was indeed found that the citrate content of milk supplemented with LABs decreased significantly after fermentation and was almost completely consumed (Figure 5). The results also confirmed the dependence of some LABs on citrate in milk fermentation. Simultaneously, our validation experiment showed that *T. thermophilus* N-1 could produce a large amount of citrate in milk under high-temperature culture conditions. Therefore, we believe that the high-temperature culture of *T. thermophilus* N-1 before milk fermentation with LABs can effectively promote the fermentation efficiency of LABs. To verify this hypothesis, we set up a treatment in which *L. fermentum* 11-1S4 was added to milk cultured with *T. thermophilus* N-1 (Figure 5). In this treatment, the absolute changes in lactose, pH and lactate were greater than those under fermentation with *L. fermentum* 11-1S4 alone. These results suggest that by producing citrate required for *L. fermentum* 11-1S4 fermentation, *T. thermophilus* N-1 may promote the fermentation of *L. fermentum* 11-1S4 in milk, acting similarly to an LAB assistant.

Conversely, LABs might assist *T. thermophilus* in becoming the most dominant thermophilic microorganism by inhibiting *Geobacillus stearothermophilus*, which is a more common Gram-positive thermophilic microorganism than *T. thermophilus* [53]. Among the four Inner Mongolia cheese samples, *G. stearothermophilus* was detected but did not surpass *T. thermophilus* as the most dominant thermophilic microorganism (Figure 1). This is probably due to the inhibitory effect of nisin secreted by LABs on Gram-positive *G. stearothermophilus* [54], which helps Gram-negative *T. thermophilus* to be preferentially enriched. Therefore, *T. thermophilus* may form a mutual relationship with LABs in the process of Inner Mongolia cheese preparation.

A starter culture may be defined as a preparation or material containing large numbers of different microorganisms, referred to as starter microorganisms, that may be added to accelerate fermentation and decrease the risk of fermentation failure [55,56]. Therefore, we conclude that *T. thermophilus* acts as a thermophilic, assistant starter microorganism in the production of Chinese Inner Mongolian cheese.

## 5. Conclusions

Chinese Inner Mongolian cheese production does not involve the artificial addition of starter microorganisms and chymosin and instead relies on the simmering of acidified milk to separate whey until milk coagulation occurs. This special process enriches thermophilic *T. thermophilus* in milk because of its lactose metabolic pathway. Although the lack of the Leloir pathway in *T. thermophilus* means that it is unable to metabolise D-galactose, resulting in lower production efficiency of lactate than is observed in LABs, it plays a role in lactose metabolism and acid production that LABs cannot perform during the simmering process in Chinese Inner Mongolian cheese fermentation. *T. thermophilus* can also produce acetate, propionate, citrate and other organic acids through unique acetate production pathways and complete propionate production pathways and TCA cycle that are absent in LABs, which affects flavour formation in Chinese Inner Mongolian cheese. Simultaneously, *T. thermophilus* can produce a large amount of citrate during the milk simmering process, which provides the carbon source substrate most needed by LABs for continuous fermentation after milk cooling, and plays a role in promoting LAB fermentation in Chinese Inner Mongolian cheese production. Therefore, *T. thermophilus* is a predominant, thermophilic, assistant starter microorganism unique to Chinese Inner Mongolian cheese.

## Figures and Tables

**Table 1:** Information on the high-throughput sequencing libraries of the bacterial 16S rRNA genes in cheese samples from Inner Mongolia.

Sample Name	Raw Reads	Clean Reads	OTU Number	Shannon Index	Simpson Index	Chao1	ACE	Library Coverage
XS	80076	72864	646	2.335	0.464	614.919	625.801	0.998
KS	73117	69204	403	1.103	0.235	398.943	409.096	0.998
ES	88637	80066	389	3.753	0.817	351.643	354.244	0.999
WS	87213	80137	413	3.884	0.816	390.818	400.815	0.999

ES, cheese sample from the Ordos grassland; XS, cheese sample from the Xilingol grassland; KS, cheese sample from the Horqin grassland; WS, cheese sample from the Ulanqab grassland. ACE, abundance-based coverage estimator.

**Table 2:** Overview of genomic features of *T. thermophilus* N-1.

Features	Chromosome	Plasmid 1	Plasmid 2
Genome topology	Circular	Circular	Circular
Genome size (bp)	1,855,628	257,206	81,373
G+C content (mol%)	69.72	69.33	67.95
Open reading frames	2328	-	-
tRNA genes	48	-	-
rRNA genes	6	-	-
sRNA genes	0	-	-
GenBank accession number	CP082323	CP082324	CP082325

G+C, Guanine + Cytosine.

## Data Availability

The data presented in this study are available on request from the corresponding author.

## Supplementary Materials

The following are available online at https://www.mdpi.com/article/10.3390/foods10122962/s1, Figure S1: COG categories of coding proteins, Table S1: The complete genome sequences of *T. thermophilus* N-1, *Lactobacillus plantarum* 10CH, *Lactococcus lactis* SD96, *Streptococcus thermophilus* NCTC 12958, *Leuconostoc mesenteroides* DSM 20484, *Lactobacillus paracasei* NJ and *Lactobacillus fermentum* MTCC 25067, and the main functional genes involved in fermentation.
