# Supplementary material for: Genomic and Metabolic Features of an Unexpectedly Predominant, Thermophilic, Assistant Starter Microorganism, Thermus thermophilus, in Chinese Inner Mongolian Cheese
Source: Foods. 2021 Dec 2;10(12):2962. doi: 10.3390/foods10122962 (PMC8700840; doi:10.3390/foods10122962)
Supplement: Supplementary file 1 [file foods-10-02962-s001.zip › foods-1387605-supplementary.pdf]

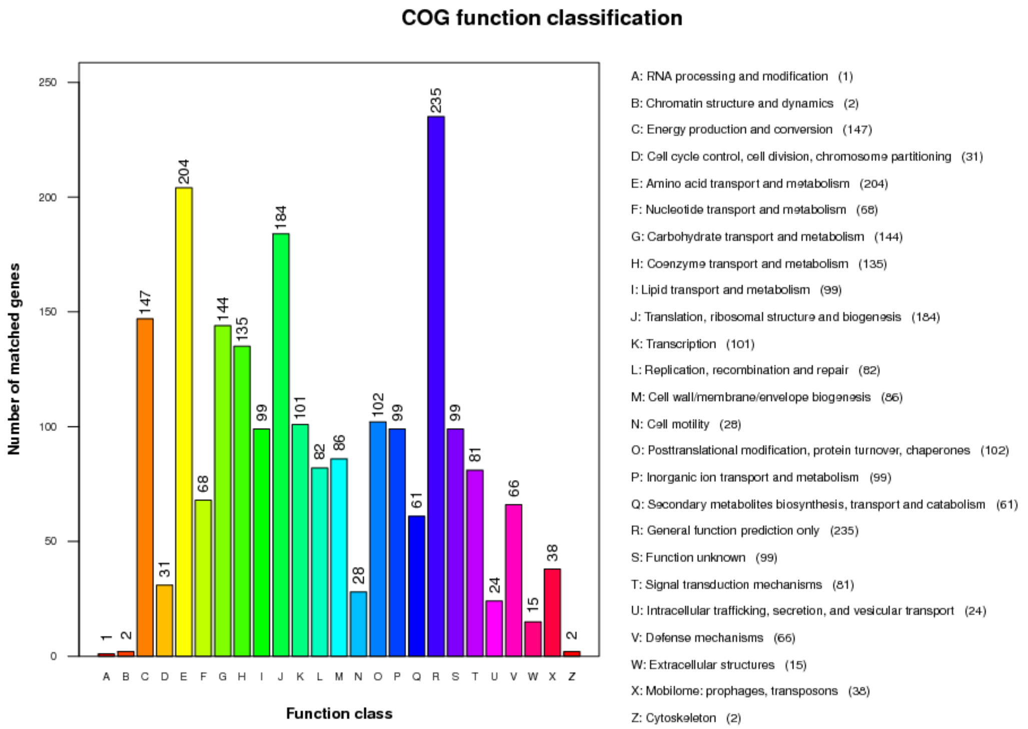

Figure S1. COG categories of coding proteins. The X-axis represents the functional classes, and the Y-axis represents the number of predicted proteins in *T. thermophilus* N-1.

Table S1 The complete genome sequences of *T. thermophilus* N-1, *Lactobacillus plantarum* 10CH, *Lactococcus lactis* SD96, *Streptococcus thermophilus* NCTC 12958, *Leuconostoc mesenteroides* DSM 20484, *Lactobacillus paracasei* NJ and *Lactobacillus fermentum* MTCC 25067, and the main functional genes involved in fermentation.

	T. thermophilus N-1	*Lactobacillus plantarum* 10CH	*Lactococcus lactis* SD96	*Streptococcus thermophilus* NCTC 12958	*Leuconostoc mesenteroides* DSM20484	*Lactobacillus paracasei* NJ	*Lactobacillus fermentum* MTCC 25067
Source	cheese	cheese	cheese	milk	cheese	yoghourt	fermented milk
Accession number	CP082323-CP082325	CP023728	CP043518-CP043528	LS483339	CP012009-CP012010	CP041944	AP017973-AP017974
Genome size	2.19	3.31	2.64	2.1	1.85	3.08	2.01
G+C content	69.72	44.5	35.3	39	38	46.4	51.1
β-galactosidase	+	+	+	+	+	-	+
glucokinase	+	+	+	+	+	+	+
L-lactate dehydrogenase	+	+	+	+	+	+	+
acetolactate synthase	+	+	+	+	+	+	+
2-oxoglutarate/2-oxoacid ferredoxin oxidoreductase	+	+	+	+	+	+	+
acetaldehyde dehydrogenase	+	+	+	-	+	+	+
acetyl-CoA synthetase	+	-	-	-	-	-	-
acetyl-CoA carboxylase/biotin carboxylase	+	+	+	+	+	+	+
enoyl-CoA hydratase	+	-	-	-	-	-	-
acyl-CoA dehydrogenase	+	+	-	-	+	-	-
